# Optimal Design of Novel Microemulsions-Based Two-Layered Dissolving Microneedles for Delivering Fluconazole in Treatment of Fungal Eye Infection

**DOI:** 10.3390/pharmaceutics14030472

**Published:** 2022-02-22

**Authors:** Phuvamin Suriyaamporn, Praneet Opanasopit, Worranan Rangsimawong, Tanasait Ngawhirunpat

**Affiliations:** 1 Pharmaceutical Development of Green Innovations Group (PDGIG), Faculty of Pharmacy, Silpakorn University, Nakhon Pathom 73000, Thailand; puvamin.su.55@ubu.ac.th (P.S.); opanasopit_p@su.ac.th (P.O.); 2 Division of Pharmaceutical Chemistry and Technology, Faculty of Pharmaceutical Sciences, Ubon Ratchathani University, Ubon Ratchathani 34190, Thailand

**Keywords:** two-layered dissolving microneedles, microemulsion, optimal mixture design, fungal keratitis, fluconazole

## Abstract

The optimal design of novel microneedles (MNs) for the ocular delivery system is necessary and useful for improving the effectiveness of medication. The objective of this study was to design and develop the optimal fluconazole (FLUZ)-microemulsions (MEs)-loaded two-layered dissolving MNs as a potential treatment for fungal eye infection. The experimental designs using the simplex-lattice design were used to select the optimal formulation. The two-layered dissolving MNs were fabricated from 3% chitosan and 20% polyvinyl alcohol (PVA) in a weight ratio of 1:4 as an outer layer and FLUZ-loaded MEs containing eugenol, tween 80, PEG400, and water as an inner layer. The physical appearance, mechanical properties, penetration ability, dissolution time, in vitro/ex vivo ocular drug delivery, and antifungal activity were evaluated. From the results, the optimal two-layered dissolving MNs exhibited good physical properties, complete insertion, minimally invasive ocular tissue, and high stability at 4 °C and 25 °C for 3 months. Moreover, the optimal two-layered dissolving MNs showed significantly higher FLUZ permeation into the ocular tissue than other formulations, while providing highly potential antifungal activity. In conclusion, the optimal MEs-loaded two-layered MNs’ formulation had appropriate properties for ocular delivery of FLUZ, resulting in an improvement of fungal keratitis treatment.

## 1. Introduction

Fungal eye infections or fungal keratitis are the major causes of ocular morbidity. This ocular disease may lead to blindness if it is not cured properly. Approximately 30,000 new cases are reported annually and at an expenditure of about $175 million in the USA healthcare sector [1]. The factors to increase ocular fungal infection are ocular trauma, overuse and/or contamination of ocular products, immunocompromised disease, and systemic fungal infections. The major sources of fungal keratitis are *Aspergillus*, *Fusarium*, *Candida,* and *Curvularia* [2]. Normally, anterior eye segment diseases such as fungal keratitis are treated by topical installation. The bioavailability of medication eye drops decreases due to the corneal barrier. The corneal epithelium is the first limiting corneal barrier in ocular drug delivery due to its lipophilicity and tight junction [3]. Moreover, nasolacrimal drainage, rapid turnover of lacrimal fluid, and the eye blinking reflex can shorten the contact time with the eye surface, leading to a decrease in the bioavailability of drugs (less than 5% bioavailable) [4,5]. Therefore, frequent instillation, increasing the drug concentration, improving contact time, or intracorneal injection are necessary. However, these techniques may lead to a decrease in patient compliance and an increase in severe systemic or local side effects [6].

Clinical pharmacology usually treats ocular fungal infections with antifungal drugs such as natamycin, amphotericin B, econazole, fluconazole, itraconazole, miconazole, ketoconazole, and voriconazole. They are used during ocular instillation, oral therapy, or subconjunctival injection [7]. Fluconazole (FLUZ), or 2-(2,4-difluorophenyl)-1,3-bis(1H-1,2,4-triazol-1-yl)-2- propanol, is categorized in the azole group of antifungal drugs; it is lipophilic and slightly soluble in water. In clinical application, FLUZ is used to treat ocular fungal infections due to its favorable safety/toxicity profile, high bioavailability, and broad-spectrum antifungal activity. For its mechanism of action, the involvement of ergosterol biosynthesis interruption is important to fluidize the fungal membranes and decrease proliferation. The concentration of FLUZ for topical treatment has been used in the range of 0.18–0.9% *w*/*w* [8,9,10]. For subconjunctival and intracorneal injection, the therapeutically effective dose of FLUZ is 0.2% *w*/*w* [11,12]. However, the limitations of antifungal drugs to provide effective treatment are fungal depth infiltrations, poor water solubility of antifungal agents, and poor water–lipid partition when permeating into the ocular barrier [13].

FLUZ is labeled as “slightly soluble in water” according to the solubility classification adopted by USP and Ph. Eur. High solubility in oils and surfactants of FLUZ indicated that this drug could be formulated as a lipid-based delivery system [14]. Lipid-based formulation, particularly microemulsions, is well known as a potential alternative approach for delivery of hydrophobic drugs and has been used as one of the most effective strategies to enhance bioavailability (bypassing the dissolution step prior to absorption). The MEs include isotropic oils, surfactants, co-surfactants, and water that produce spontaneous oil-in-water microemulsions immediately under gentle stirring. Several advantages of MEs have been reported such as improved drug loading, increased bioavailability, and thermodynamic stability. Moreover, this system is suitable for ocular drug delivery because the small droplet size of MEs can enhance ocular drug bioavailability by either prolonging drug release and/or improving drug penetration through the ocular tissues. MEs are safe and biocompatible for the treatment of ocular diseases with better penetrability and have a suitable pharmaceutical effect [15,16,17,18,19,20]. However, passive penetration of MEs only might be unable to deliver drugs through the ocular tissue at the therapeutically effective dose. The combination of passive microemulsion and a minimally invasive technique should be optimized and investigated.

Microneedles (MNs) are a micron-scale technology (60 to 1000 μm in height) that is minimally invasive to ocular tissue. The mechanism of MNs on ocular application is the creation of aqueous conduits on the cornea, bypassing the corneal barrier, and improving the bioavailability of ophthalmic drugs. The polymeric microneedles are fabricated from biocompatible or biodegradable polymers that cause minimal damage to ocular tissue, which allows it to heal within 24 h and provides non-medical waste after complete dissolution [21]. Dissolving MNs that completely dissolved faster than hydrogel-forming MNs have been used to incorporate several micro- or nanoparticle formulations into polymeric MNs, such as liposomal-amphotericin-B-loaded dissolving MNs [22], cubosome-rapamycin-loaded dissolving MNs [23], nanosuspension-curcumin-loaded dissolving MNs [24], and nanocrystals-itraconazole-loaded dissolving MNs [25]. This combination provides a beneficial option for improving treatment of many diseases and enhancing both solubility and penetrability of loaded drugs. However, the ocular delivery device from microemulsion-loaded microneedles as a two-layered dissolving microneedle has not been optimized and evaluated yet.

To improve the effectiveness of fungal eye infection treatment by the antifungal drug, FLUZ, the combination of ocular delivery systems has to be designed and optimized for enhancing the solubility and stability of the drug as well as the ocular penetrability. Therefore, the purpose of this experiment was to design and develop the optimal FLUZ-MEs-loaded two-layered dissolving MNs’ formulations as a potential treatment for fungal eye infection. A mixture of experimental designs using simplex-lattice designs was used to design and select the optimal MEs’ formulation before loading in two-layered dissolving MNs. The optimal MEs’ formulation was characterized by size, PDI, zeta potential, pH, centrifugation test, drug content, and an in vitro ocular permeation study. The two-layered dissolving MNs that were fabricated from biocompatible or biodegradable polymers such as chitosan and polyvinyl alcohol (PVA) polymer as a first layer microneedle were mixed in appropriate ratios. After that, the optimal FLUZ-MEs-loaded two-layered dissolving MNs were tested in terms of their physical appearance under a scanning electron microscope (SEM) and confocal microscopy, mechanical strength properties and insertion force by a texture analyzer, depth of insertion by confocal microscopy, dissolution time, drug content, in vitro/ex vivo permeation of drug into the eyes, drug remaining in ocular tissue, antifungal activity, and a stability test.

## 2. Materials and Methods

### 2.1. Materials

Fluconazole was purchased from the Tokyo Chemical Industry (Tokyo, Japan). Eugenol was obtained from Bruno Court (Grasse, France). Chitosan (low molecular weight; 50–190 kDa), polyvinyl alcohol (PVA 99+% hydrolyzed; 89–98 kDa), PEG400, and Tween 80 were bought from Sigma-Aldrich (Dorset, UK). Gantrez^®^ S-97 (Poly-methyl vinyl ether-alt-maleic acid; MW = 1500 Da) was purchased from Ashland Inc. (Surrey, UK). Hank’s Balanced Salt Solution was prepared from D-glucose (anhydrous) 1.0 g/L, NaCl 8.0 g/L, NaHCO_3_ 0.35 g/L, Na_2_HPO_4_ (anhydrous) 0.04788 g/L, KCl 0.4 g/L, KH_2_PO_4_ (anhydrous) 0.06 g/L, MgSO_4_ (anhydrous) 0.09767 g/L, and CaCl_2_ dihydrate 0.185 g/L. All chemical agents were analytical reagent grade.

### 2.2. Construction of Pseudo-Ternary Phase Diagrams

MEs consist of three components: oil, S_mix_ (surfactant and co-surfactant), and water. Based on the highest FLUZ solubility, each pseudo-ternary phase diagram was constructed by eugenol as the oil, Tween 80 as the surfactant, and PEG400 as the cosurfactant with different ratios of surfactants to co-surfactants (S_mix_) as 1:1, 1:2, 2:1, and 3:1. The mixture containing the oil, surfactant, and co-surfactant was mixed with the weight ratio of the oil to the S_mix_ at 1:9, 2:8, 3:7, 4:6, 5:5, 6:4, 7:3, 8:2, 9:1, respectively. The aqueous titration method was done to each weight ratio of oil and S_mix_ under moderate stirring. The diluted mixtures were directly observed visually for any phase separation. The mixtures with a transparent, clear, or slightly bluish appearance were considered as the microemulsion region of the pseudo-ternary phase diagram [26,27]. The pseudo-ternary phase diagram was constructed using OriginPro^®^ 2020 graphing and data analysis software (trial version).

### 2.3. Formulation of FLUZ Loaded MEs

The 10% *w*/*w* of FLUZ was dissolved in eugenol using a vortex mixer. Selected amounts of S_mix_ (Tween 80 and PEG400) and water by computer design were added to the mixtures. These mixtures were mixed and warmed to 40 °C for 10 min by a water bath to ensure complete mixing. The characterizations of FLUZ-loaded MEs were evaluated.

### 2.4. Development and Optimization of FLUZ Loaded MEs Using I-Optimal Design

The mixture component of MEs was designed based on a three-component system: the oil (X_1_: eugenol), the S_mix_ (X_2_: Tween 80 and PEG 400), and the water (X_3_). In this study, the concentration of oil (6.25–25.00% *w*/*w*), S_mix_ (66.25–87.50% *w*/*w*), and water (6.25–25.00% *w*/*w*) were selected as input factors. The output factors for evaluation of the ME formulation were droplet size (Y_1_), PDI (Y_2_), drug content (Y_3_), and % of permeation at 8 h (Y_4_). The concentrations of the three components were changed by computer design simultaneously, but the total concentration was kept at 100% [28]. The Design-Expert^®^ version 11 software generated 14 batches of microemulsion for the evaluation of the relation between input factors and output factors as shown in Table 1.

### 2.5. Characterization of FLUZ-Loaded MEs

#### 2.5.1. Droplet Size, PDI, and Zeta Potential Measurement

The droplet size, PDI, and zeta potential of FLUZ-loaded MEs were measured by photon correlation spectroscopy (Zetasizer Nano Series, Malvern Instruments, Malvern, UK). Light scattering was monitored at 25 °C at a 90° angle.

#### 2.5.2. pH Value

The pH value of MEs was determined using a pH meter (Laquatwin Horiba, Kyoto, Japan) to determine the appropriate formulations for the eyes [29].

#### 2.5.3. Centrifugation Test

Each MEs’ formulation was centrifuged at 4000 rpm for 15 min to determine whether the system showed signs of creaming or phase separation [30].

#### 2.5.4. Drug Content Determination

The FLUZ concentration in each sample was determined by HPLC. The chromatographic separation was performed using a Zorbax Eclipse XDB-C18 reverse-phase column (250 × 4.6 mm, 5 µm pore size) (Agilent, Santa Clara, CA, USA). The FLUZ-loaded MEs were dissolved in an appropriate volume of methanol. After that, the mixtures are filtered through a cellulose filter (0.45 μm). The conditions of FLUZ analysis using isocratic elution were 45% *v*/*v* methanol: 55% *v*/*v* ultrapure water. Then, 20 µL of each sample was injected into an HPLC system with a flow rate of 1 mL/min, the wavelength of detection at 260 nm, and an oven temperature at 25 ± 2 °C. The retention time of FLUZ was 4.9 min [31].

#### 2.5.5. Percent of Permeation at 8 h

The delivery of FLUZ-loaded MEs through porcine corneal tissue was examined by a hemisphere-shaped vertical Franz diffusion cell. The receptor compartment was filled with 4 mL of PBS pH 7.4, maintained at a temperature of 37 °C, and continuously stirred by a magnetic stirrer bar. Each MEs formulation (0.5 mL) was added to the donor chamber. The samples (300 μL) from the receptor compartment were collected at 8 h. An equal volume of PBS pH 7.4 was refilled to maintain a volume in the receptor compartment. The concentrations of FLUZ were analyzed by HPLC in triplicate. The percentage of permeation at 8 h was calculated by the following Equation (1).

(1) $$\%\mathrm{Permeation}\;\mathrm{at}\;8\;h=\frac{\mathrm{Amount}\;\mathrm{of}\;\mathrm{drug}\;\mathrm{in}\;\mathrm{receptor}\;\mathrm{compartment}\;\mathrm{after}\;8\;h\;(\text{µ}g)}{\mathrm{Amount}\;\mathrm{of}\;\mathrm{initial}\;\mathrm{drug}\;\mathrm{in}\;\mathrm{donor}\;\mathrm{compartment}\;(\text{µ}g)}\times \;100$$

### 2.6. Fabrication of FLUZ-MEs-Loaded Two-Layered Dissolving MNs

**Figure 1:**
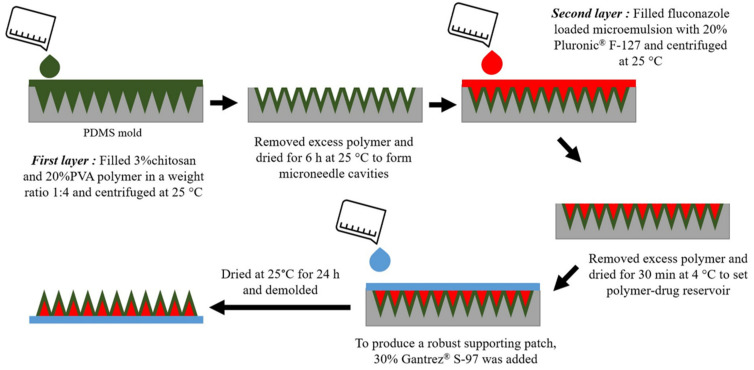
Schematic diagram of fabrication of FLUZ-MEs-loaded two-layered dissolving MNs.

Two-layered dissolving MNs were fabricated by the simple micro-molding method as presented in Figure 1. The polydimethylsiloxane (PDMS; Blueacre technology, Ireland) molds have a microneedle density of 11 × 11, conical-shaped needles, with an average height of 600 μm, 300 μm width at base, and 300 μm interspacing. In this study, the first layer of MNs was prepared from five different weight ratios of 3% *w*/*w* chitosan and 20% *w*/*w* PVA (1:1, 1:2, 1:3, 1:4, and 1:5) [32]. The stock polymer solution of 3% *w*/*w* chitosan was prepared in 1% acetic acid solution and 20% *w*/*w* PVA was dissolved in water at 80 °C in the water bath. Briefly, the 200 mg of polymer mixture was cast into PDMS micromolds as a first layer and centrifuged (ALC, PK121R, Caerphilly, UK) at 4000 rpm, 25 °C. for 30 min to fulfill the polymer mixture in the cavities of the molds and to eliminate bubbles. Afterward, the excess of the polymer mixture on the top of the PDMS molds was removed and dried at room temperature for 6 h. Then, 200 mg of optimal FLUZ MEs suspended in 20% Pluronic^®^ F-127 as a second layer was poured into the PDMS micromolds and centrifuged. The excess FLUZ MEs was removed. MNs in the micromold were kept in the refrigerator at 4 °C for 30 min to set the Pluronic^®^ F-127. To produce a robust supporting patch, 30% Gantrez^®^ S-97 was added and centrifuged at 2000 rpm, 4 °C, for 10 min. Finally, the FLUZ-MEs-loaded two-layered dissolving MNs were dried at 25 °C for 24 h and gently peeled out of the PDMS molds. The FLUZ-MEs-loaded two-layered dissolving MNs were collected and kept in the desiccator before further characterization.

For the control MNs formulation, FLUZ-suspension-loaded MNs were fabricated. A 1.27% *w*/*w* of FLUZ suspension was loaded into a polymer mixture of 3% *w*/*w* chitosan and 20% *w*/*w* PVA (1:4 weight ratio). This mixture was cast into PDMS micromolds and centrifuged at 4000 rpm, 25 °C, for 30 min. The control MNs were dried at 25 °C for 24 h and gently peeled out of the PDMS molds.

### 2.7. The Physical Appearance of MEs-Loaded Two-Layered Dissolving MNs and FLUZ Content

The physical appearance of FLUZ-MEs-loaded two-layered dissolving MNs was evaluated under SEM (Mira TC, Czech Republic) at a beam voltage of 10.0 kV with 30× and 130× magnification. The MNs were measured in height, width, and interneedle spacing. To confirm two-layered dissolving MNs, the first layer was loaded with 1% fluorescein sodium (FS) as a water-soluble phase and the second layer was loaded with 0.5% rhodamine b as an oil phase. A confocal laser scanning microscope (CLSM; OLYMPUS FV1000, Tokyo, Japan) was used to visualize this MN.

To perform the drug loading, the FLUZ-MEs-loaded two-layered dissolving MNs were dissolved in 10 mL of methanol. The sample solution was centrifuged at 14,000 rpm for 30 min at 25 °C and the supernatant was filtered by a 0.45 µm nylon syringe filter (Vertical^®^, Bangkok, Thailand) before being analyzed by HPLC. The percentage of loading efficiency (%LE) was calculated by following Equation (2) [33].

(2) $$\%\mathrm{Loading}\;\mathrm{efficiency}=\frac{\mathrm{Actual}\;\mathrm{amount}\;\mathrm{of}\;\mathrm{FLUZ}\;(\mathrm{mg})}{\mathrm{Initial}\;\mathrm{Amount}\;\mathrm{of}\;\mathrm{FLUZ}\;(\mathrm{mg})}\times \;100$$

### 2.8. Preparation of Porcine Eyeballs and Corneal Tissue

Porcine eyes have been widely used in human eye studies because the ocular histology and water content of porcine eyes are similar to the human eye [34,35]. The fresh porcine eyeballs were received from the local slaughterhouse (Nakhon Pathom, Thailand). The porcine eyeballs were immediately used within 24 h after being received or frozen at −20 °C in a sealed container with phosphate buffer saline (PBS) pH 7.4 until further use within 3 months [36]. In the ocular tissue preparation, the porcine eyeballs were thawed in a water bath at 37 °C, and then adherent muscle tissue was removed. The corneal tissue at the anterior segment of the eye was cut circumferentially away from the limbus around 1 cm and soaked in PBS pH 7.4 at 37 °C for 30 min before being used in this experiment.

### 2.9. Mechanical Strength and Insertion Force of FLUZ-MEs-Loaded Two-Layered Dissolving MNs

A texture analyzer (TA.XT plus, Stable Microsystems, Godalming, UK) connected with a 5 kg load cell was used to determine the mechanical strength of the MNs’ formulations using compression mode. The first-layered MNs’ formulations fabricated from various weight ratios of 3% *w*/*w* chitosan and 20% *w*/*w* PVA (1:1, 1:2, 1:3, 1:4, and 1:5) were evaluated. Briefly, MNs’ patches were gently attached to the 1 cm cylinder stainless probe (P/1KSS) in a downward direction. The stainless probe was moved downward at a speed of 1 mm/s to make the MNs attach to flat stainless steel, and the force was continuously increased until it reached a displacement of 600 μm. Then, the stainless probe was moved upward at a speed of 1 mm/s. Afterward, the highest mechanical strengths of the first-layered MNs were selected to fabricate two-layered dissolving MNs. The two-layered dissolving MNs with/without loaded FLUZ MEs were tested for their mechanical strength. The insertion force of the dissolving MNs with/without loaded FLUZ MEs were tested on the porcine corneal tissues that were fixed on a hemisphere dental wax (half-shaped eyeball). The compression mode was used to evaluate the MNs as previously described. The force versus displacement curves were plotted.

### 2.10. Determination of Complete Insertion and Depth of Insertion

The ability of FLUZ-MEs-loaded two-layered dissolving MNs to complete insertion on corneal tissue was tested on the artificial membrane polymeric film (Parafilm M^®^; Bemis NA, Neenah, WI, USA). This MN array was placed on the 5 layers of polymeric film (mimics the corneal thickness around 650 µm) [37] and then used the insertion force from the previous study for 30 s. The number of visible dots on the polymeric film surface were counted under a Dino-Lite digital microscope (Hsinchu, Taiwan). The insertion ability of the MNs was calculated by the following Equation (3).

(3) $$\mathrm{Percentage}\;\mathrm{of}\;\mathrm{complete}\;\mathrm{insertion}=\frac{\mathrm{Number}\;\mathrm{of}\;\mathrm{visible}\;\mathrm{dots}}{\mathrm{Number}\;\mathrm{of}\;\mathrm{microneedles}}\times \;100$$

To evaluate the depth of insertion, the artificial membranes from the previous study were removed from each artificial membrane layer and the last remaining visible dot under a digital microscope was observed to calculate the depth. Each artificial membrane layer’s thickness is 0.13 mm [37]. Moreover, the fluorescein-sodium-loaded two-layered MNs were applied on the porcine corneal tissue for 3 min. After removing the MN patch, the applied corneal tissues were washed with PBS pH 7.4 and the depth of insertion was evaluated under a CLSM. The corneal tissue at different penetration depths was performed by top view confocal micrographs [21]. The cross sectioning of the applied corneal tissues that were fixed with 4% formaldehyde solution (24 h) were embedded in frozen section media and then cross sectioned by cryostat (CM1850, Leica Biosystems, Nußloch, Germany) at −35 °C (10 μm thickness). The samples were immediately observed under a fluorescence microscope (Nikon^®^ T-DH, Tokyo, Japan) [38].

### 2.11. Dissolution Times of FLUZ-MEs-Loaded Two-Layered Dissolving MNs

The dissolution time of FLUZ-MEs-loaded two-layered dissolving MNs after being applied on corneal tissue was determined. The prepared corneal tissue was fixed on a hemisphere dental wax (half-shape eyeball) and dropped with 0.5 mL of PBS pH 7.4 as tears. This MNs’ formulation was inserted into corneal tissue with an insertion force for 30 s. At predetermined times of 0, 1, 2, and 3 min, the MNs were withdrawn and observed under a digital microscope [21].

### 2.12. In Vitro Ocular Permeation of FLUZ-MEs-Loaded Two-Layered Dissolving MNs

The ocular permeation of FLUZ-MEs-loaded two-layered dissolving MNs were performed on the porcine corneal tissues. An adapted Franz diffusion cell containing a hemisphere-shaped compartment to fit and support the curvature of the corneal tissues was used for ocular permeation. The receptor compartment was filled with 4 mL of PBS pH 7.4, maintained at a temperature of 37 °C, and continuously stirred by a magnetic stirrer bar. Each formulation containing 1.27% FLUZ was added to the donor chamber. The samples (300 μL) from the receptor compartment were collected at predetermined times including 0, 0.25, 0.5, 1, 2, 4, 6, 8, 12, and 24 h. An equal volume of PBS pH 7.4 was refilled to maintain a volume in the receptor compartment. The concentrations of FLUZ were analyzed by HPLC in triplicate.

The data from ocular permeation profiles that can explain the behavior of how the drug was delivered across corneal tissue were the cumulative amount of FLUZ per area (Q_24_/A), Flux (*J*), lag time (t_lag_), the diffusion coefficient (K_d_), and the permeability coefficient (K_p_). The *J* and t_lag_ can be calculated on the slope of the graph Q_24_/A versus time and interception on the time-axis graph, respectively. The K_d_ and K_p_ were calculated by the following Equations (4) and (5).

(4) $$\mathrm{Kd}=\frac{\mathrm{Thickness}\;\mathrm{of}\;\mathrm{corneal}\;\mathrm{tissue}\;2\;(h2)}{6\;\times \;\mathrm{tlag}\;}$$

(5) $$\mathrm{Kp}=\frac{J}{\mathrm{Concentration}\;\mathrm{of}\;\mathrm{FLUZ}\;\mathrm{in}\;\mathrm{donor}\;(\mathrm{Cd})}$$

### 2.13. The %FLUZ Retained in Corneal Tissues

After the ocular permeation study, the corneal tissues were cleaned with PBS pH 7.4, sliced into small pieces using surgical scissors, and homogenized using a probe sonicator (60 Hz) in 5 mL methanol for 20 min. Afterward, the solution with small pieces of corneal tissues was centrifuged at 4000 rpm for 20 min. Finally, the supernatant was analyzed by HPLC. The percentage of FLUZ retained in corneal tissues was calculated following Equation (6).

(6) $$\%\mathrm{FLUZ}\;\mathrm{retained}\;\mathrm{in}\;\mathrm{corneal}\;\mathrm{tissues}=\frac{\mathrm{Amount}\;\mathrm{of}\;\mathrm{FLUZ}\;\mathrm{in}\;\mathrm{corneal}\;\mathrm{tissue}}{\mathrm{Amount}\;\mathrm{of}\;\mathrm{FLUZ}\;\mathrm{loading}}\;\times \;100$$

### 2.14. Ex Vivo Ocular Drug Delivery of FLUZ-MEs-Loaded Two-Layered Dissolving MNs

The FLUZ-MEs-loaded two-layered dissolving MN formulation was applied on the cornea of the whole porcine eyeball. To set mimetic intraocular pressure, the catheter connected to a bottle of Hank’s Balanced Salt solution was inserted into the vitreous chamber via the optic nerve, and a bottle was placed at a height of 5 cm from the experimental area [21,39,40]. After applying MNs, porcine eyeballs were wrapped with tissue paper soaked in PBS and kept in an incubator at 37 °C. The 300 μL samples were collected from the aqueous humor behind the cornea using a 27G hypodermic needle with a syringe at 0, 0.5, 1, 2, 4, 6, and 8h. The amount of FLUZ was analyzed by HPLC and plotted as the percent of permeation at 8 h versus time.

### 2.15. Antifungal Activity of FLUZ-MEs-Loaded Two-Layered Dissolving MNs

The antifungal activity of the FLUZ-MEs-loaded two-layered dissolving MNs was studied against *Candida albicans* (ATCC 10231) that is associated with fungal keratitis. In the agar diffusion method, 1 mL of activated fungal growth (3 × 10^7^ CFU/mL, 1U OD600) was spread on the sabouraud dextrose agar (SDA) plate [41]. Two-layered dissolving MNs with/without 1.27% FLUZ-MEs were placed in the SDA plate with needles inside the nutrient agar. Untreated fungal lawns were used as the negative control. The 1.27% FLUZ suspension, eugenol, 1.27% FLUZ in eugenol, optimal MEs, and 1.27% FLUZ-loaded optimal MEs were used as positive controls. The treated fungal plates were incubated at 37 °C for 24 h. The diameter of the inhibition zone was measured using a digital microscope after 24 h of incubation and the percent of zone inhibition was calculated.

For ex vivo antifungal activity, an excised porcine cornea was incubated in an antibiotic-free medium overnight at 37 °C before starting the experiment. An amount of 50 µL of *C. albicans* (3 × 10^7^ CFU/mL, 1U OD600) was injected using a 26-gauge needle in the corneal stroma and incubated in the antibiotic-free medium for 24 h at 37 °C. After 24 h, the cornea was rinsed with PBS pH 7.4 and treated with 1.27% FLUZ in suspension, eugenol, optimal MEs, and MEs-loaded two-layered dissolving MNs. The negative control was the infected corneas without any treatment. A sterile metal ring was placed on the infected cornea to fill 200 μL of treated liquid formulations. The FLUZ-MEs-loaded two-layered dissolving MNs were inserted into the infected cornea. After 5 min of application, the formulations were withdrawn and the infected corneas were further incubated for 24 h at 37 °C. Afterward, the infected corneas were homogenized using a probe sonicator. The supernatants were serially diluted and spread onto SDA plates for 24 h at 37 °C. The colonies were counted and their colony-forming units per mL (CFU/mL) were calculated as shown in the following Equation (7).

(7) $$\mathrm{CFU}/\mathrm{mL}=\frac{\mathrm{Number}\;\mathrm{of}\;\mathrm{colonies}\;\times \;\mathrm{dilution}\;\mathrm{factor}}{\mathrm{Volume}\;\mathrm{of}\;\mathrm{culture}\;\mathrm{plated}}$$

### 2.16. Stability Study Test

FLUZ-MEs-loaded two-layered dissolving MNs were packed into an aluminum zipper pouch with silica gel and stored at 4 °C, 25 °C, and 40 °C for 3 months. The physical appearance, machinal strength, and drug content were evaluated.

### 2.17. Statistics Analysis

Each study was replicated three times and presented as means ± standard deviation (SD). The Anderson–Darling test was used to test the normality of data. The comparison of the two groups was calculated by the independent *t*-test. Statistical analysis of the calculated mechanical strength of MNs and in vitro ocular drug delivery were performed by one-way ANOVA test with a post hoc Tukey’s test. SPSS^®^ software version 19 (SPSS Inc., Chicago, IL, USA) was used. The *p*-value less than 0.05 was considered statistically significant.

## 3. Results and Discussion

### 3.1. Construction of Pseudo-Ternary Phase Diagrams

From the preliminary study, the solubility of FLUZ exhibited the highest solubility in eugenol, indicating that oil potentially increased the solubility of lipophilic FLUZ. Moreover, oil is an important component to formulate the MEs and can improve ocular drug delivery by increasing the flexibility of the ocular barrier at the epithelium. For surfactants that act as a thin forming film at the interface, they decrease the ME size, increase the stability of emulsion, and enhance ocular permeation activity by partitioning into the ocular barrier and disrupting the structural organization of the lipid ocular barrier (epithelium). FLUZ presented the highest solubility in Tween 80 among the surfactants investigated. Another part of MEs is that the co-surfactant can increase the interfacial fluidity and create void spaces between the interface for water penetration, providing spontaneous emulsification [42]. In this study, FLUZ presented high solubility in PEG400 with safety for ocular tissue among the co-surfactant investigated. Thus, eugenol, Tween 80, and PEG400 were selected for formulating MEs based on the solubility using ternary phase diagrams. The data of FLUZ solubility are presented in the Supplementary Materials (Table S1).

**Figure 2:**
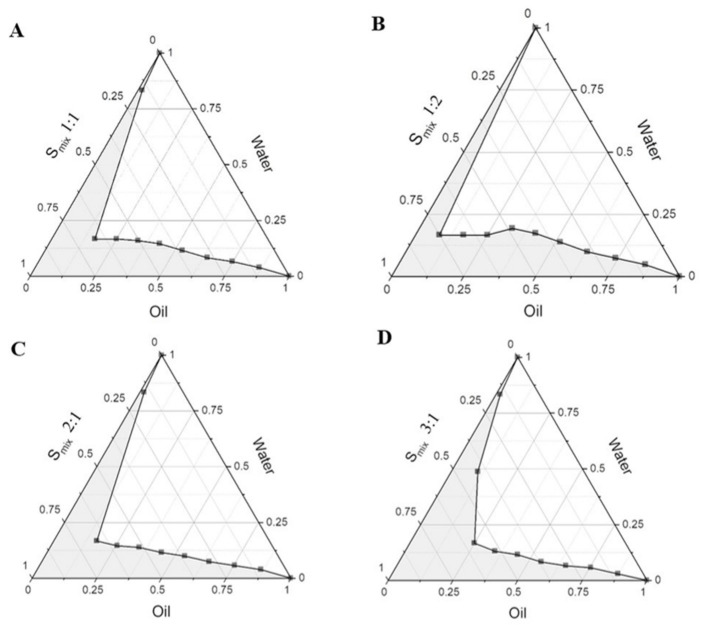
Pseudo-ternary phase diagram of eugenol, Tween 80, and PEG400 containing various S_mix_ ratios: (**A**) 1:1 with area 33.43%; (**B**) 1:2 with area 31.98%; (**C**) 2:1 with area 34.35%; and (**D**) 3:1 with area 40.48%.

The construction of pseudo-ternary phase diagrams was used to determine the translucent region of MEs. Four pseudo-ternary phase diagrams for MEs with various weight ratios of S_mix_ are presented in Figure 2. The highest existence region of the MEs was discovered in the S_mix_ ratio at 3:1 and around 40.48%. The results from the pseudo-ternary phase diagrams revealed that increasing the weight ratio of the surfactant enlarged the MEs existence region.

### 3.2. Development and Optimization of FLUZ-Loaded MEs Using I-Optimal Design

The component of selected MEs (S_mix_ ratio 3:1) was optimized by I-optimal mixture design. The levels selected for mixture design were oil 6.25–25%, S_mix_ 66.25–87.50%, and water 6.25–25%, creating a total of 14 experimental batches. The results of output factors such as droplet size (Y_1_), PDI (Y_2_), drug content (Y_3_), and % of permeation at 8 h (Y_4_) are shown in Table 1.

**Figure 3:**
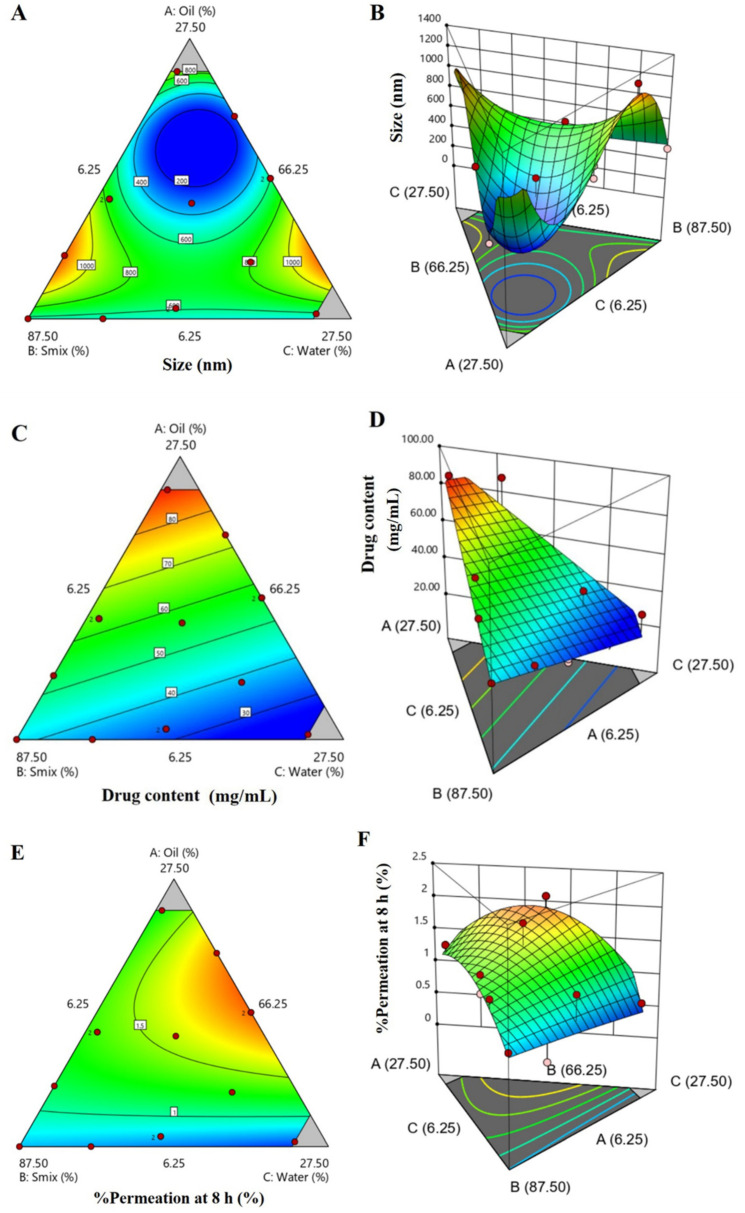
2D contour plot and 3D response surface area of (**A**,**B**): size; (**C**,**D**): drug content; and (**E**,**F**): % of permeation at 8 h of FLUZ-loaded MEs formulations.

In this study, the globule size of FLUZ-loaded MEs was found to be around 179.13 to 1322.67 nm, which the analysis from the 2D contour plot and the 3D response surface area represent in Figure 3A,B. When the ratio of oil increased together with the decreasing ratio of S_mix_ and water, the globule size of MEs was decreased in the appropriate ratio range (dark blue color) for PDI that was found to be around 0.30 to 0.95. PDI is used to measure the broadness of molecular weight distribution, at which the appropriate PDI of MEs should not be more than 0.30 [43]. Thus, experimental batch No. 6 showed a suitable PDI for MEs (Table 1).

The drug content of FLUZ-loaded MEs was found to be around 26.53 to 90.28 mg/mL, which the analysis from the 2D contour plot and the 3D response surface area are represented in Figure 3C,D. Increasing the concentration of oil increased the drug content in a linear manner (red color), while increasing the Smix and water decreased the amount of the drug content, respectively. The percent of permeation at 8 h of FLUZ-loaded MEs was found to be around 0.44 to 2.08%. As shown in Figure 3E,F, the percent of permeation at 8 h of FLUZ-loaded MEs analysis from the 2D contour plot and the 3D response surface area represent that increasing the ratio of oil and water increased the percent of permeation of FLUZ-loaded MEs (red color). This effect could be expanded by the multi-layered structure of the cornea. From anterior to posterior, there is the epithelium, Bowman’s layer, stroma, Descemet’s membrane, and endothelium. Stroma is the hydrophilic barrier’s nature to transport water-soluble molecules into the cornea, whereas the other structures are the barrier’s lipoidal nature [44]. MEs composed of oil and water can undergo this multi-layered structure of the cornea, and the surfactant and co-surfactant could improve the cornea barrier’s fluidity. ANOVA analysis of size (Y_1_), PDI (Y_2_), drug content (Y_3_) and % of permeation at 8 h (Y_4_) are presented in the Supplementary Materials (Tables S2–S5).

To optimize the FLUZ-loaded MEs formulations, the Design-Expert^®^ version 11 software was used to analyze and summarize the data with suitable criteria as presented in Table 2. The optimal FLUZ-loaded MEs formulations were 20.546% oil (eugenol) + 67.70% S_mix_ (3:1) (Tween80 and PEG400) + 11.76% water with acceptable and excellent desirability of 0.83. The desirability was a tool to evaluate the multi-response optimization value, in which the acceptable and excellent desirability was between 0.8 and 1, referring to the good quality of the formulation [45]. The predicted value and actual value of output factors were tested statistically using *t*-test to confirm the selected model. As illustrated in Table 3, the selected model of FLUZ-loaded MEs’ formulations was reliable with no significant difference between the predicted and the actual value. In addition, the optimal MEs’ formulations showed appreciated PDI around 0.30 ± 0.02, neutral zeta potential 0.012 ± 0.001 mV, and pH 6.91 ± 0.10, suggesting that the small globule size could pass through the gap junction of the cornea barrier and not be attached from the positive charge on the cornea [44]. The pH of optimal MEs was 7.11 ± 1.5, showing a suitable formulation to apply on the ocular tissue [46]. After the centrifugation test, this ME was physically stable and did not have any phase separation.

### 3.3. The Physical Appearance of MEs-Loaded Two-Layered Dissolving MNs and FLUZ Content

**Figure 4:**
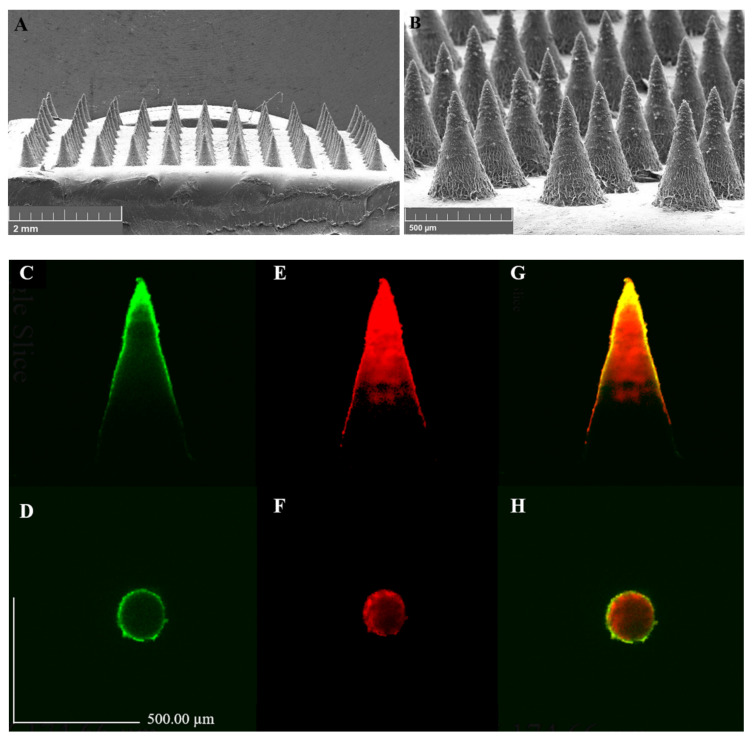
Appearance of FLUZ-MEs-loaded two-layered dissolving MNs: SEM images (magnification (**A**) 30× and (**B**) 130×) and confocal images in side view and top view of (**C**,**D**) loaded FS (green color); (**E**,**F**) loaded rhodamine b (red color); and (**G**,**H**) both first layer FS and second layer rhodamine b.

As shown in Figure 4, the physical appearance of FLUZ-MEs-loaded two-layered dissolving MNs was the conical-shaped needle tips (11 × 11 array) with 581.83 ± 10.58 μm in height, 300.03 ± 1.51 μm in base width, and 300.10 ± 0.12 μm in interneedle spacing. This indicated the appropriate morphology to penetrate into the cornea barrier with minimal invasion because the cornea thickness is approximately 550–600 μm [47,48]. Moreover, the confocal fluorescence images showed that polymer-film-loaded FS (water phase) as the first layer MNs can separate from the FLUZ-MEs-loaded rhodamine b (oil phase) as second layer MNs.

To confirm the therapeutic dose of FLUZ, the amount of FLUZ in MEs-loaded two-layered dissolving MNs was quantified. The drug content was 12.69 ± 0.32 mg per 121 needles or 0.10 ± 0.01 mg per needle, indicating that the therapeutic dose of FLUZ in this formulation was near to or higher than topical/intracorneal injection treatment (0.18–0.9% *w*/*w*) [8,9,11]. Moreover, the percentage of loading efficiency (%LE) was 86.21 ± 2.89%. These results indicated that the outer layer of the polymer film can enclose FLUZ MEs within two-layered dissolving MNs and have less interaction with the drug before being released.

### 3.4. Mechanical Strength and Insertion Force of FLUZ-MEs-Loaded Two-Layered Dissolving MNs

**Figure 5:**
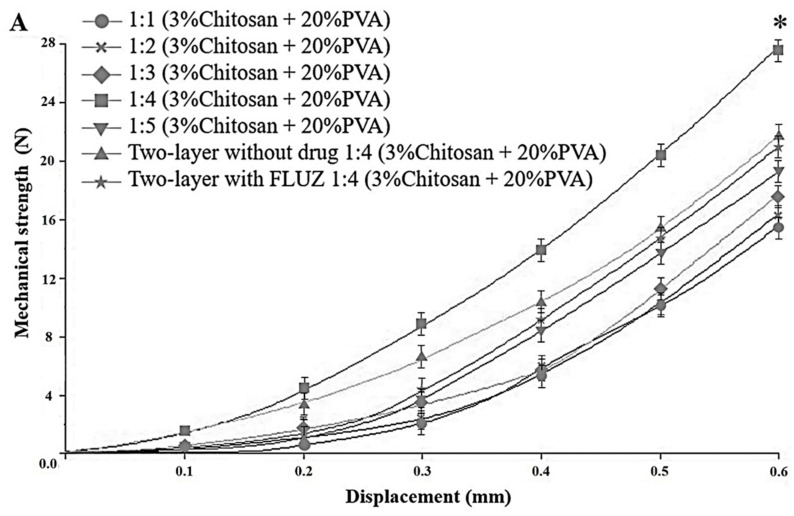

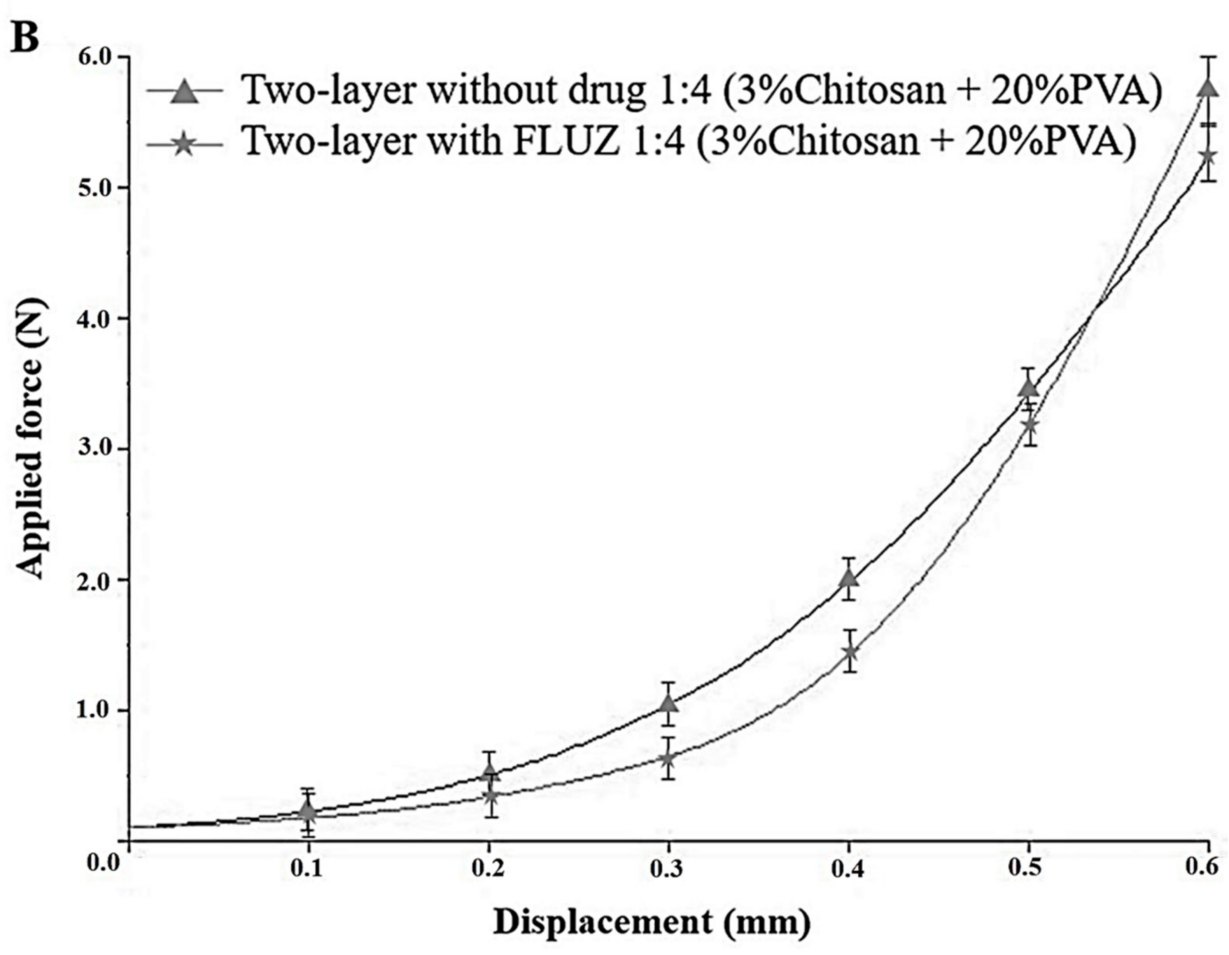
(**A**) Mechanical strength and (**B**) insertion force of optimal FLUZ-MEs-loaded two-layered dissolving MNs. * indicates significant differences from others (*p* < 0.05).

The ability to penetrate the cornea of MNs is an important characteristic of MNs to withstand the applied forces. Figure 5A showed the mechanical strength of 3% chitosan and 20% PVA MNs in a weight ratio of 1:4 was significantly higher than in other formulations. The functional groups such as -NH_2_ and -OH without charge can be involved in hydrogen bonding, but the charged moieties (NH_3_^+^ and OH^−^) of the functional groups can contribute to ionic bonding only. [32]. However, increasing the weight ratio of PVA by more than five decreased the hardness of MNs, suggesting that the excess PVA (non-interaction) softened the MNs. Therefore, a weight ratio of 1:4 (3% chitosan and 20% PVA) was chosen to fabricate the first or outer layer of two-layered dissolving MNs. The mechanical strengths of two-layered dissolving MNs with and without FLUZ were 21.57 N ± 1.04 and 20.85 ± 1.09 N, respectively, indicating that FLUZ loaded into the inner layer (1.27% *w*/*w*) did not significantly change the mechanical strength of the two-layered dissolving MNs. To completely insert into the cornea (600 µm thick), the estimated insertion forces were 5.70 ± 0.51 N/121 array or 0.05 N per needle in control and 5.18 ± 0.49 N/121 array or 0.04 N per needle in optimal formulation (Figure 5B), referring to the appropriate insertion forces to use as an ocular device patch (0.05–0.15 N) with minimally invasive corneal tissue [22,37,38].

### 3.5. Determination of Complete Insertion and Depth of Insertion in Ocular Tissues

**Figure 6:**
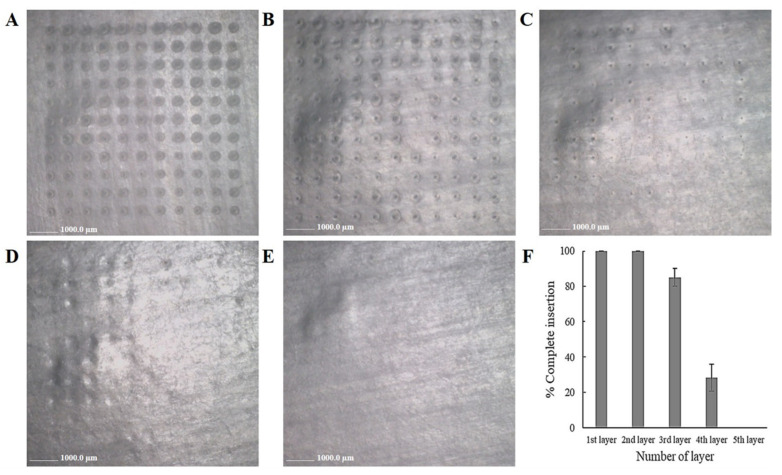
Images of complete insertion into artificial membrane (Parafilm M^®^) of FLUZ-MEs-loaded two-layered dissolving MNs: (**A**) 1st layer; (**B**) 2nd layer; (**C**) 3rd layer; (**D**) 4th layer; (**E**) 5th layer; and (**F**) percentage of complete insertion versus number of layers.

To overcome the cornea barrier, the complete insertion of MNs was tested. However, it is difficult to observe the visible dots on cornea tissue. Thus, the determination of complete insertion of MNs was carried out on an artificial membrane (Parafilm M^®^) with the same thickness of corneal tissue. The five layers of artificial membrane, being 650 µm thick (~0.13 µm thick per each layer), were applied to the FLUZ-MEs-loaded two-layered dissolving MNs with insertion force (5.70 ± 0.51 N/121 array), showing the complete insertion (100%) in the first and second layers, while the third and fourth layers had 85.00 ± 5.00% and 28.33% ± 7.64%, respectively as shown in Figure 6. Although the fifth layer was not inserted, the insertion into the fourth layer (around 520 µm in depth) represented the ability to bypass the corneal thickness (around 500–800 µm) [44].

**Figure 7:**
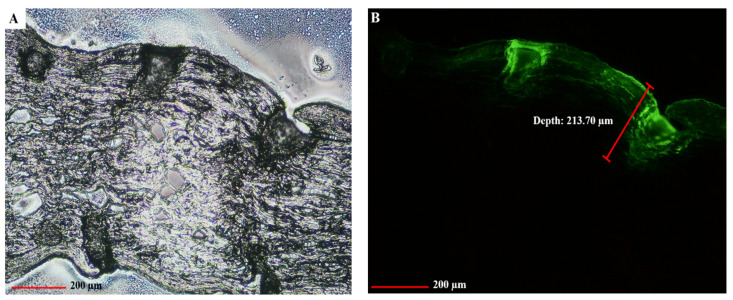

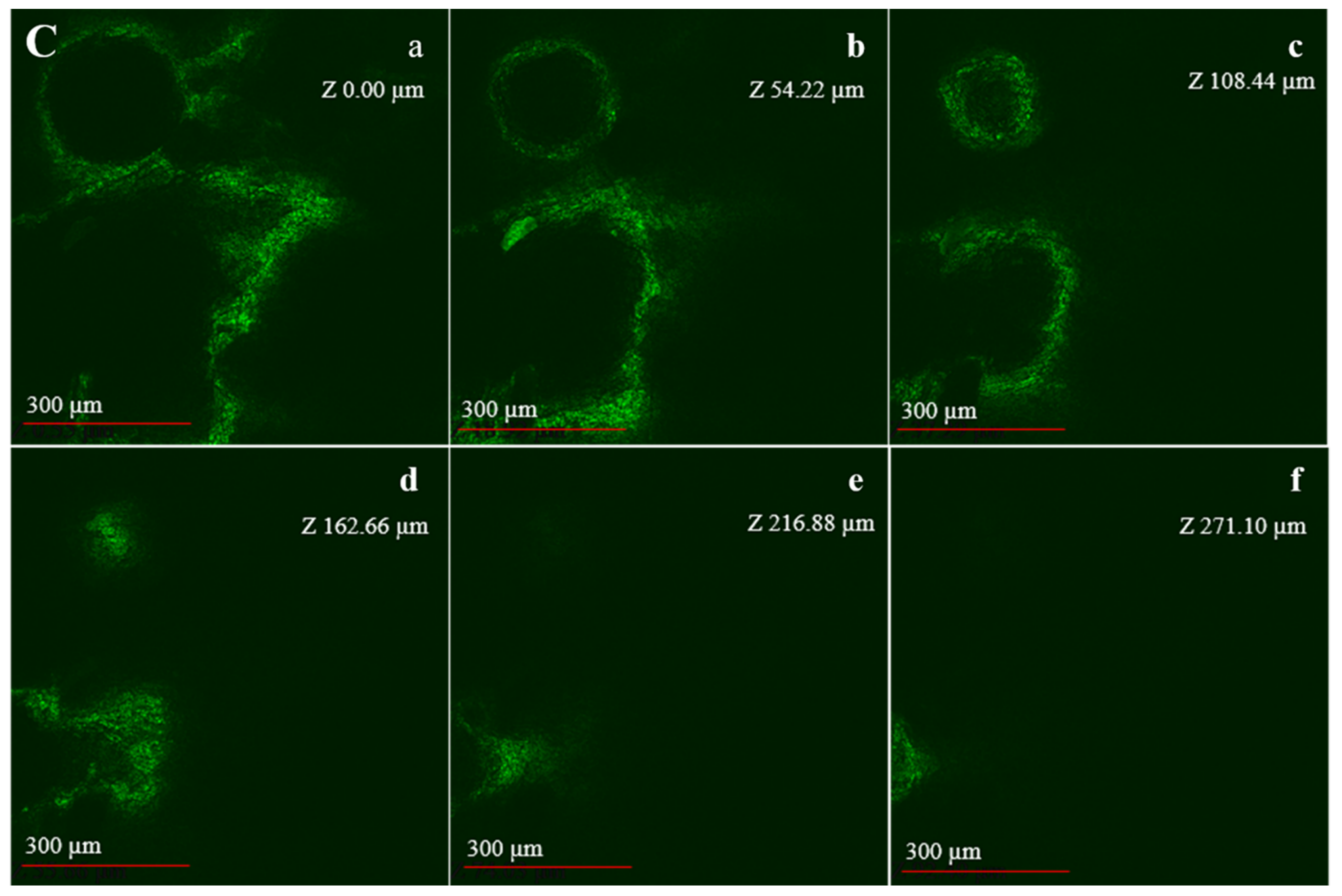
Depth of insertion of FLUZ-MEs-loaded two-layered dissolving MNs in corneal tissues: (**A**) bright field of cross-section image; (**B**) fluorescence of cross-section image (10× objective lens); and (**C**) x-y plane CLSM image at difference penetration depths inside the corneal tissue: (**a**) 0.00 μm, (**b**) 54.22 μm, (**c**) 108.44 μm, (**d**) 162.66 μm, (**e**) 216.88 μm and (**f**) 271.10 μm.

To confirm this depth of insertion, FS-loaded two-layered MNs were applied on porcine corneal tissues and the depth of insertion was observed under a fluorescence microscope and a CLSM. As shown in Figure 7, the observed depths of the MNs’ insertion from a cross-section image and x-y plane CLSM images were 213.70 μm and 271.10 μm. The results of the insertion depth were different from previous tests in artificial membranes (Figure 6) because the elastic structure of corneal tissues is highly resistant to deformation. However, this result indicated that the FLUZ-MEs-loaded two-layered dissolving MNs were successfully inserted into the corneal tissue by creating the microchannel into this tissue to deliver FLUZ into the deeper layer.

In addition, the dissolution times of dissolving MNs were an important test to design and develop the dissolving MNs’ formulation for ocular application, in which rapid dissolution could increase the patient complaint. The FLUZ-MEs-loaded two-layered dissolving MNs completely dissolved within 3 min after being applied to the corneal tissue. This result indicated that this MNs’ formulation had an appropriate dissolution time. The images of MNs’ dissolution times are presented in the Supplementary Materials (Figure S1).

### 3.6. In Vitro Ocular Permeation of FLUZ-ME- Loaded Two-Layered Dissolving MNs and FLUZ Remaining in Corneal Tissue

**Figure 8:**
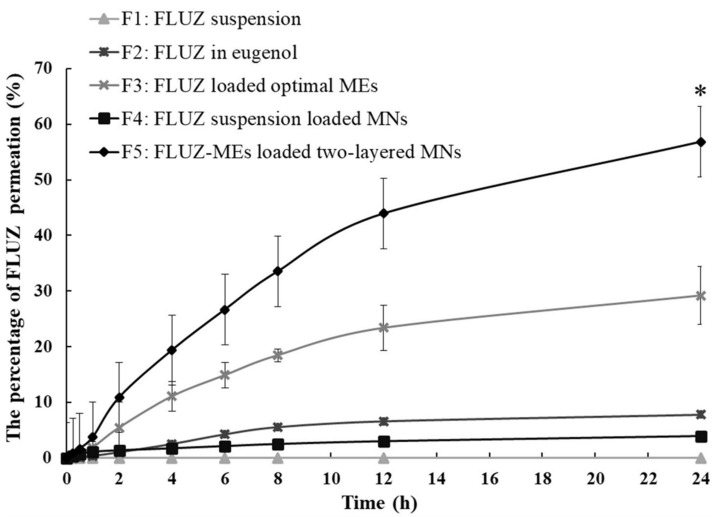
The percentage of FLUZ permeation in 24 h through porcine corneal tissues from different formulations. * indicates significantly higher than other formulations (*p* < 0.05).

As shown in Figure 8, the percentages of FLUZ permeation in 24 h through the corneal tissue were in the order: FLUZ-MEs-loaded two-layered dissolving MNs (56.84 ± 2.61%) > FLUZ-loaded optimal MEs (29.20 ± 5.20%) > FLUZ in eugenol (7.78 ± 0.30%) > FLUZ-suspension-loaded MNs (3.95 ± 0.23%) > FLUZ suspension (0.00 ± 0.00%). The FLUZ-MEs-loaded two-layered dissolving MNs had a significantly higher FLUZ permeation than other formulations, while the ocular permeation parameters, *J* and Q_24_/A values, were also significantly higher than others (Table 4). In addition, the lag time of MNs’ formulations was short at around 0.18–0.19 h because MNs could create a microchannel into the corneal tissue and the entrapped FLUZ was released faster than with topical formulations. The short lag time of MNs’ formulations-loaded-FLUZ resulted in a high diffusion coefficient (K_d_). Moreover, FLUZ-MEs-loaded two-layered dissolving MNs improved the permeability coefficient (K_p_) of FLUZ into the corneal tissue.

FLUZ is a moderate lipophilic drug with a low-log partition coefficient (log *P* 0.58) [49]; thus, FLUZ suspension was not allowed to partition into the cornea. For the formulation of FLUZ dissolved in oil, the ocular permeation was still at a low value, due to high drug accumulation in the epithelium barrier of the cornea. The corneal barrier structure was composed of a hydrophilic and a lipophilic structure (Fat–Water–Fat); thus, MEs’ formulations could increase the ocular permeation profile. This suggested that the enhancing mechanism of MEs might be the result of increasing the oil–aqueous phase ratio of lipophilic drugs across the cornea structure, adapting the cornea structure and decreasing the droplet size of MEs [42,50]. However, only passive penetration of MEs might not be able to deliver drugs through the ocular tissue at the therapeutically effective dose. To enhance the ocular delivery of FLUZ, the minimally invasive MNs were used to bypass this corneal barrier structure. The single-layer MNs containing FLUZ suspension showed a low ocular permeation profile because FLUZ was not completely dissolved in the water compartment. Consequently, the FLUZ-MEs-loaded two-layered dissolving MNs performed a high ocular permeation profile, suggesting the combination mechanism of the bypassing corneal barrier structure and improving the permeability of lipophilic drugs.

The percentage of FLUZ remaining in the corneal tissue after 24 h of FLUZ-MEs-loaded two-layered MNs was significantly higher than that of other formulations (Table 4), indicating that the needles of FLUZ-MEs-loaded two-layered dissolving MNs deposited in the corneal tissue after creating microchannels. This was the large quantity of FLUZ reservoir before delivery through the eye.

### 3.7. Ex Vivo Ocular Drug Delivery of FLUZ-MEs-Loaded Two-Layered Dissolving MNs

**Figure 9:**
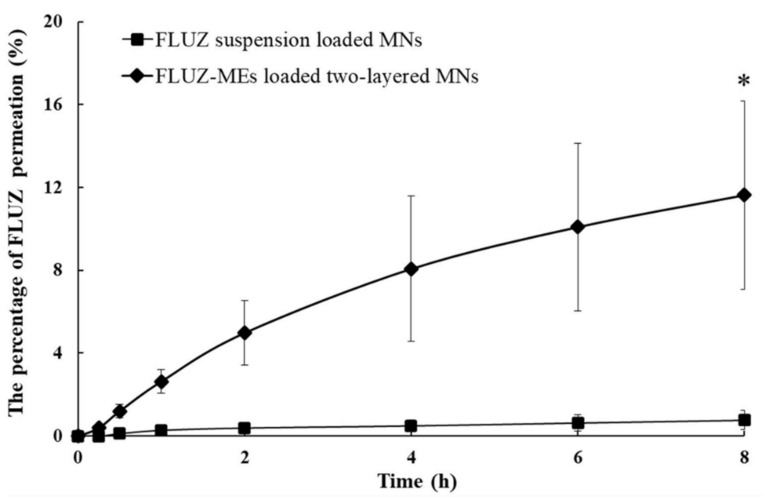
Ex vivo ocular drug delivery of FLUZ-MEs-loaded two-layered dissolving MNs compared with FLUZ-suspension-loaded MNs. * indicates significantly higher than other formulations (*p* < 0.05).

The ex vivo ocular FLUZ delivery was tested in the whole porcine eyeballs that have ocular histology and water content similar to the human eye [34]. The % of FLUZ permeation in 8 h of FLUZ-MEs-loaded two-layered dissolving MNs was 11.63 ± 4.54% and significantly higher than FLUZ-suspension-loaded MNs, represented in Figure 9. For the ocular permeation profiles, the lag time, *J*, Q_24_/A, K_d_, and K_p_ of FLUZ-MEs-loaded two-layered dissolving MNs was 0.16 ± 0.02 h, 0.24 ± 0.10 mg/cm^2^/h, 1.88 ± 0.58 mg/cm^3^, 3.79 ± 0.48 × 10^−3^ cm^2^/h, and 19.08 ± 8.15 × 10^−3^ cm^2^/h, respectively. The ex vivo ocular FLUZ delivery in 8 h was lower than the in vitro study because the dissolving MNs might be dissolved on the cornea before complete insertion. Moreover, FLUZ was eliminated via the vascular endothelium after crossing the cornea barrier. However, the % of FLUZ retained in the corneal tissue was 30.01 ± 3.18%, indicating that the drug in two-layer MNs’ formulations was highly retained in the corneal tissue as a large reservoir drug matrix. These results exhibited the potential formulation to deliver antifungal drugs for the treatment of fungal keratitis.

### 3.8. Antifungal Activity of FLUZ-MEs-Loaded Two-Layered Dissolving MNs

**Figure 10:**
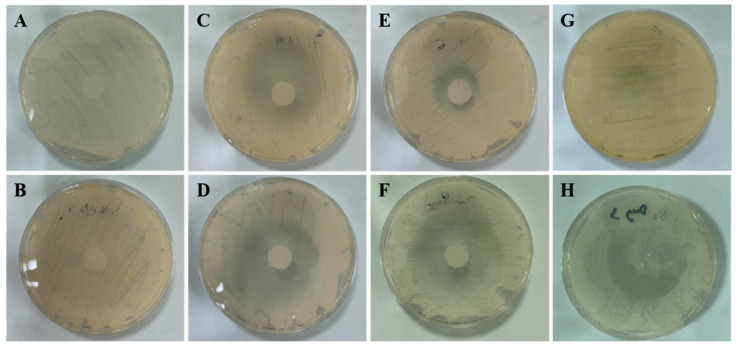
Antifungal activity of agar diffusion assay results using *C. albicans*: (**A**) control; (**B**) FLUZ suspension; (**C**) eugenol; (**D**) FLUZ in eugenol; (**E**) optimal MEs; (**F**) FLUZ-loaded optimal MEs; (**G**) two-layered MNs; and (**H**) FLUZ-MEs-loaded two-layered MNs.

As illustrated in Figure 10, the insertion of FLUZ-MEs-loaded two-layered dissolving MNs into an SDA agar plate showed a diameter zone of inhibition similar to FLUZ in eugenol and FLUZ-loaded optimal MEs at the same concentration of the drug. This result indicated that the FLUZ-MEs were completely released from the two-layer MNs and showed potential antifungal activity. In the case of FLUZ suspension, the insolubility of FLUZ in water provided a low percent zone of inhibition because the ability to penetrate into the *C. albicans*’ plasma membrane was reduced. The mechanism of the azoles group is based on blocking the synthesis of ergosterol and the cytochrome P450 dependent enzyme in the plasma membrane; thus, the delivery of the azole drug into the target site presented the inhibition of fungal growth [51]. Moreover, eugenol had potent antifungal activity against *C. albicans* from the phenolic hydroxyl group in the eugenol structure to disrupt the plasma membrane [52]. Therefore, eugenol provided a synergic effect with FLUZ to kill *C. albicans* by enhancing FLUZ solubility and antifungal activity. The data on the % of zone of inhibition are reported in Table 5.

**Figure 11:**
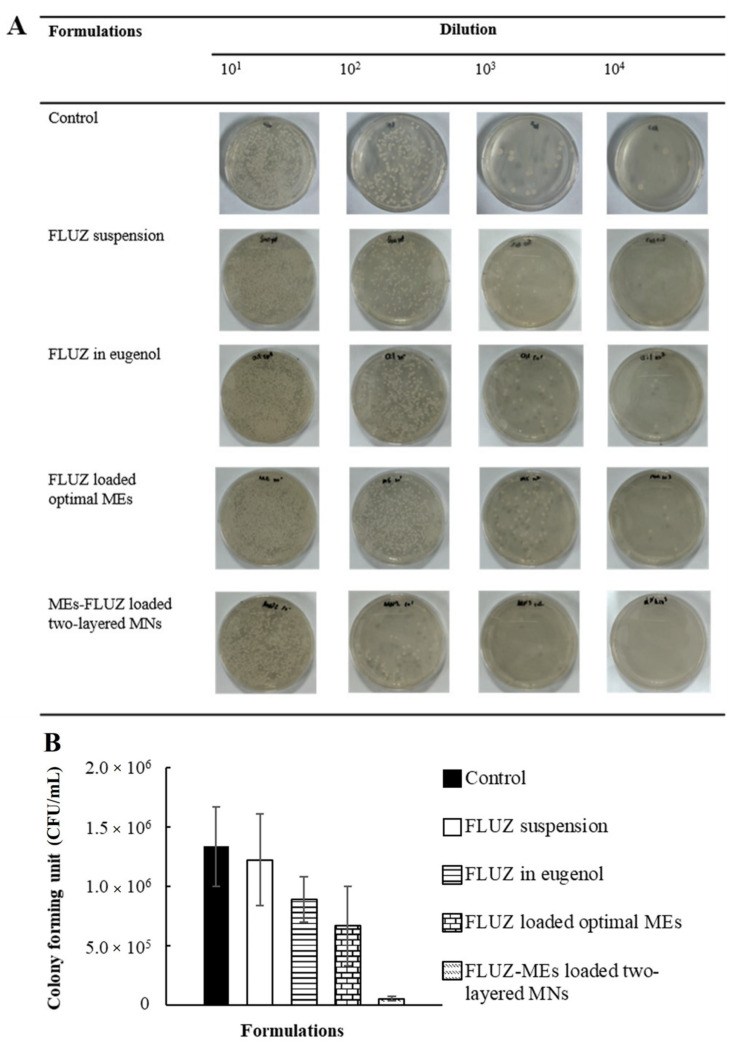
Ex vivo antifungal activity in excised porcine corneal tissues. (**A**) *C. albicans* colonies formed after 24 h of diluted corneal homogenate on SBA plate with different FLUZ formulations. (**B**) Colony-forming units after 24 h with different FLUZ formulations. Each formulation is representative of triplicate trials. * indicates a significant difference than other formulations (*p* < 0.05).

To evaluate antifungal activity in the corneal tissue, FLUZ formulations were applied on the infected fungal cornea. As shown in Figure 11, FLUZ-MEs-loaded two-layered dissolving MNs inserted into corneal tissue showed the least colony-forming unit (5.56 ± 1.92 × 10^4^ CFU/mL), indicating that FLUZ-MEs-loaded two-layered dissolving MNs provided sufficient concentration of antifungal activity at the fungal infection area and the deeper corneal tissue. Whereas, the topical formulations could not penetrate into the deeper layer of the corneal tissue and the result was a decrease in the efficacy of antifungal drugs for the treatment of fungal keratitis.

### 3.9. Stability Test

**Figure 12:**
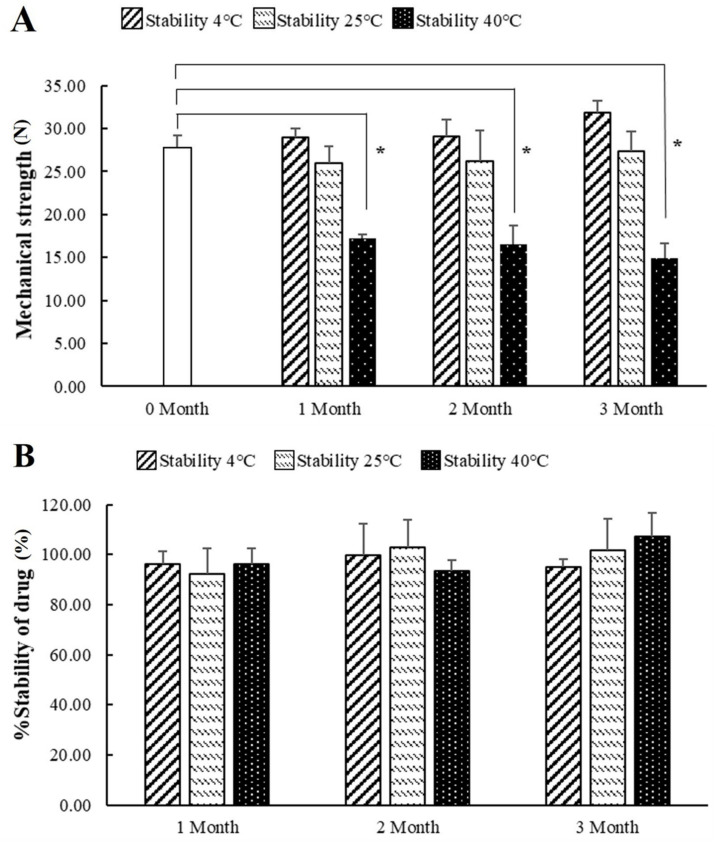
Stability of FLUZ-MEs-loaded two-layered MNs at 4 °C, 25 °C, and 40 °C for 0, 1, 2, and 3 months. (**A**) The mechanical strength and (**B**) % of stability of the drug. All data are represented in means ± SD with triplicate trials. * indicates significantly lower than 0 month (*p* < 0.05).

The stability of FLUZ-MEs-loaded two-layered MNs was studied for 1, 2, and 3 months at 4 °C, 25 °C, and 40 °C in an aluminum zipper pouch with silica gel to control humidity. As shown in Figure 12, the physical appearance was not changed from the initial aspect. The mechanical strength did not significantly differ between 0 and 3 months at 4 °C and 25 °C, while the higher temperature (40 °C) significantly decreased the mechanical strength by affecting the hardness of the polymer. At the lower temperature (4 °C), the mechanical strength slightly increased due to polymer shrinkage. The stability of the drug content did not significantly change between 1 and 3 months. The results presented that FLUZ-MEs-loaded two-layered MNs had a high physical and chemical stability at 4 °C and 25 °C over 3 months.

## 4. Conclusions

Novel two-layered dissolving MNs were successfully optimized and fabricated for ocular delivery of FLUZ. The outer layer was fabricated from 3% chitosan and 20% PVA polymer in a weight ratio of 1:4, while the inner layer was optimal MEs’ loaded with FLUZ. The optimal FLUZ-MEs-loaded two-layered MNs showed sufficient physical properties, mechanical strength, and penetration ability with minimal invasion on corneal tissue. These MNs have potential use for intracorneal FLUZ delivery with complete dissolution within 3 min. Importantly, FLUZ-MEs-loaded two-layered MNs showed highly effective antifungal activity in the *C. albicans*-infected corneal tissue. Moreover, the stability of these MNs was suitable at 4 °C and 25 °C over a 3-month period. Therefore, the optimal FLUZ-MEs-loaded two-layered MNs’ formulation had appropriate properties for ocular delivery of FLUZ, resulting in an improvement of fungal keratitis treatment.

## Figures and Tables

**Table 1:** Experimental batches of FLUZ-loaded MEs and output factors using I-optimal design.

Batches	Input Factor1 A: Oil (% *w*/*w*)	Input Factor2 B: S_mix_ (% *w*/*w*)	Input Factor3 C: Water (% *w*/*w*)	Output Factor1 Size (nm)	Output Factor2 PDI	Output Factor3 Drug Content (mg/mL)	Output Factor4 %Permeation at 8 h (%)
1	16.89	66.25	16.86	635.03	0.42	41.82	1.86
2	15.33	77.60	7.06	603.20	0.61	64.08	1.37
3	6.63	68.38	25.00	625.80	0.62	31.67	0.63
4	7.04	77.39	15.57	668.00	0.65	28.43	0.66
5	25.00	68.33	6.67	970.83	0.95	90.28	1.36
6	21.61	66.25	12.14	179.13	0.30	87.92	1.64
7	16.89	66.25	16.86	634.37	0.60	71.31	2.08
8	15.33	77.60	7.06	725.70	0.58	63.08	1.09
9	7.04	77.39	15.57	522.10	0.79	26.53	1.15
10	6.25	82.57	11.18	577.00	0.68	38.72	0.44
11	6.25	87.50	6.25	504.80	0.56	42.10	0.81
12	10.55	70.72	18.73	632.97	0.50	46.38	0.97
13	15.01	72.36	12.63	505.47	0.77	42.90	1.91
14	11.05	82.70	6.25	1322.67	0.72	58.55	1.28

**Table 2:** Criteria for optimized FLUZ-loaded MEs formulation.

Factors	Criteria	Solutions	Desirability
A: Oil (% *w*/*w*)	is in range	20.54% *w*/*w*	0.83
B: S_mix_ (% *w*/*w*)	is in range	67.70% *w*/*w*	
C: Water (% *w*/*w*)	is in range	11.76% *w*/*w*	
Size (nm)	Minimize	119.85 nm	
PDI	none		
Drug content (mg/mL)	Maximize	74.13 mg/mL	
% of permeation at 8 h (%)	Maximize	1.78%	

**Table 3:** Predicted value and actual value of output factors from optimized FLUZ-loaded MEs formulation.

Results	Output Factors		
	Size (nm)	Drug Content (mg/mL)	% of Permeation at 8 h (%)
Predicted value	119.85 ± 0.00	74.13 ± 0.00	1.78 ± 0.00
Actual value	121.22 ± 9.01	73.58 ± 0.54	1.57 ± 0.22
*t*-test (*p*-value)	0.81	0.15	0.17

**Table 4:** Ocular permeation profile and drug retained in corneal tissue of FLUZ.

Ocular Permeation Profile ^#^	Formulations				
	FLUZ Suspension	FLUZ in Eugenol	FLUZ Loaded Optimal MEs	FLUZ-Suspension-Loaded MNs	FLUZ-MEs-Loaded Two-Layered MNs
Lag time (h)	0.00 ± 0.00	0.60 ± 0.12	0.74 ± 0.30	0.18 ± 0.06	0.19 ± 0.08
*J* (mg/cm^2^/h)	0.00 ± 0.00	0.12 ± 0.01	0.39 ± 0.03	0.11 ± 0.02	0.70 ± 0.13 *
Q_24_/A (mg/cm^2^)	0.00 ± 0.00	1.26 ± 0.05	4.72 ± 0.84	0.64 ± 0.04	9.19 ± 0.33 *
K_d_ (×10^−3^, cm^2^/h)	0.00 ± 0.00	1.02 ± 0.21	0.89 ± 0.31	3.66 ± 1.47	3.56 ± 1.20
K_p_ (×10^−3^, cm^2^/h)	0.00 ± 0.00	9.17 ± 0.70	30.93 ± 2.06	8.60 ± 1.52	55.17 ± 10.36 *
% FLUZ retained in cornea (%)	0.00 ± 0.00	27.72 ± 4.33	14.94 ± 1.34	16.90 ± 3.28	38.45 ± 3.27 *

^#^ Ocular permeation profile: *J*, flux; Q_24_/A, cumulative amount of FLUZ at 24 h per area; K_d_, diffusion coefficient; K_p_, permeability coefficient. * indicates significantly higher than other formulations (*p* < 0.05).

**Table 5:** The percentage of zone inhibition obtained from agar diffusion assay. (N = 3).

Formulations ^#^	A	B	C	D	E	F	G	H
% of zone inhibition (%)	0.00 ±0.00	0.00 ±0.00	50.39 ±3.02	58.82 ±5.88	36.08 ±1.36	60.00 ±2.04	3.92 ±1.70	61.96 ±5.80

**^#^** Formulation code: (A) control; (B) FLUZ suspension; (C) eugenol; (D) FLUZ in eugenol; (E) optimal MEs; (F) FLUZ-loaded optimal MEs; (G) two-layered MNs; and (H) FLUZ-MEs-loaded two-layered MNs.

## Data Availability

Not applicable.

## Supplementary Materials

The following are available online at https://www.mdpi.com/article/10.3390/pharmaceutics14030472/s1, Figure S1: The dissolution times of MEs-FLUZ-loaded two-layered dissolving MNs in corneal tissues at (A) 0 min, (B) 1 min, (C) 2 min, and (D) 3 min; Table S1: Solubility of FLUZ in various oils, surfactants, and co-surfactants; Table S2: ANOVA analysis of size (Y_1_); Table S3: ANOVA analysis of PDI (Y_2_); Table S4: ANOVA analysis of drug content (Y_3_); and Table S5: ANOVA analysis of % of permeation at 8 h (Y_4_).
