# Supplementary material for: Optimal Design of Novel Microemulsions-Based Two-Layered Dissolving Microneedles for Delivering Fluconazole in Treatment of Fungal Eye Infection
Source: Pharmaceutics. 2022 Feb 22;14(3):472. doi: 10.3390/pharmaceutics14030472 (PMC8951261; doi:10.3390/pharmaceutics14030472)
Supplement: Supplementary file 1 [file pharmaceutics-14-00472-s001.zip › pharmaceutics-1604041-supplementary.pdf]

pharmaceutics

MDPI

Supplementary Materials: Optimal Design of Novel Micro-emulsions-Based Two-Layered Dissolving Microneedles for Delivering Fluconazole in Treatment of Fungal Eye Infection

Table S1. Solubility of FLUZ in various oils, surfactants and co-surfactants.

Solvent	Solubility (mg/mL)	Solvent	Solubility (mg/mL)	Solvent	Solubility (mg/mL)
Oils		Surfactants		Co-Surfactants	
**Eugenol***	**198.92 ± 11.21**	Tween 20	20.81 ± 2.66	Ethanol	68.52 ± 2.00
Oleic acid	52.17 ± 0.42	**Tween 80***	**45.96 ± 2.03**	**PEG 400***	**64.29 ± 4.04**
IPM	18.76 ± 4.03	Span 20	21.07 ± 0.26	Propylene glycol	39.85 ± 2.85
Peppermint oil	9.35 ± 2.30	Span 80	31.27 ± 0.48	Glycerol	39.26 ± 0.33
Limonene	9.15 ± 5.00	Cremophor RH40	7.73 ± 1.15		
MC-TG	5.00 ± 2.26	Labrasol®	34.90 ± 2.50		
Orange oil	13.60 ± 0.68	Labrafac™ PG	8.27 ± 5.15		
Wintergreen oil	23.47 ± 0.18				

Table S2: ANOVA analysis of size (Y_1)

Source	Sum of squares	Degree of free-	Mean square	F-value	*p*-value	Inference
**Cubic Model**	719923.94	6	119987.32	6.44	< 0.05	Significant
Linear Mix-ture	88025.52	2	44012.76	2.36	0.16	
AB	116665.06	1	116665.06	6.26	< 0.05	
AC	203989.12	1	203989.12	10.95	< 0.05	
AB(A-B)	308463.92	1	308463.92	16.55	< 0.05	
AC(A-C)	371682.67	1	371682.67	19.94	< 0.05	
**Residual**	130451.63	7	18635.95			
Lack of Fit	112304.88	4	28076.22	4.64	0.12	Not significant
Pure Error	18146.75	3	6048.92			
**Cor Total**	850375.56	13				

Regression coefficient: R^2 = 0.85, Adjusted R^2 = 0.71, Predicted R^2 = 0.69

Table S3: ANOVA analysis of PDI (Y_2)

Source	Sum of squares	Degree of free-	Mean square	F-value	*p*-value	Inference
**Model**	0.14	3	0.05	2.28	0.14	not significant
Linear Mixture	0.03	2	0.01	0.63	0.55	
AC	0.11	1	0.11	5.58	0.04	
**Residual**	0.20	10	0.02			
Lack of Fit	0.17	7	0.02	2.61	0.23	not significant
Pure Error	0.03	3	0.01			
**Cor Total**	0.34	13				

Regression coefficient: R^2 = 0.41, Adjusted R^2 = 0.23, Predicted R^2 = -0.63

Table S4: ANOVA analysis of drug content (Y_3)

Source	Sum of squares	Degree of free-	Mean square	F-value	*p*-value	Inference
**Linear Model**	4517.74	2	2258.87	24.12	< 0.0001	significant
Linear Mixture	4517.74	2	2258.87	24.12	< 0.0001	
**Residual**	1029.96	11	93.63			
Lack of Fit	592.81	8	74.10	0.51	0.80	not significant
Pure Error	437.14	3	145.71			
**Cor Total**	5547.70	13				
Regression coefficient: R^2 = 0.81, Adjusted R^2 = 0.78, Predicted R^2 = 0.72						

Table S5: ANOVA analysis of %Permeation at 8 h (Y_4)

Source	Sum of squares	Degree of free-	Mean square	F-value	*p*-value	Inference
**Quadratic Model**	2.60	3	0.87	11.15	0.0016	significant
Linear Mixture	1.81	2	0.90	11.62	0.0025	
AC	0.79	1	0.79	10.20	0.0096	
**Residual**	0.78	10	0.08			
Lack of Fit	0.59	7	0.08	1.38	0.4305	not significant
Pure Error	0.18	3	0.06			
**Cor Total**	3.38	13				

Regression coefficient: R^2 = 0.77, Adjusted R^2 = 0.70, Predicted R^2 = 0.63

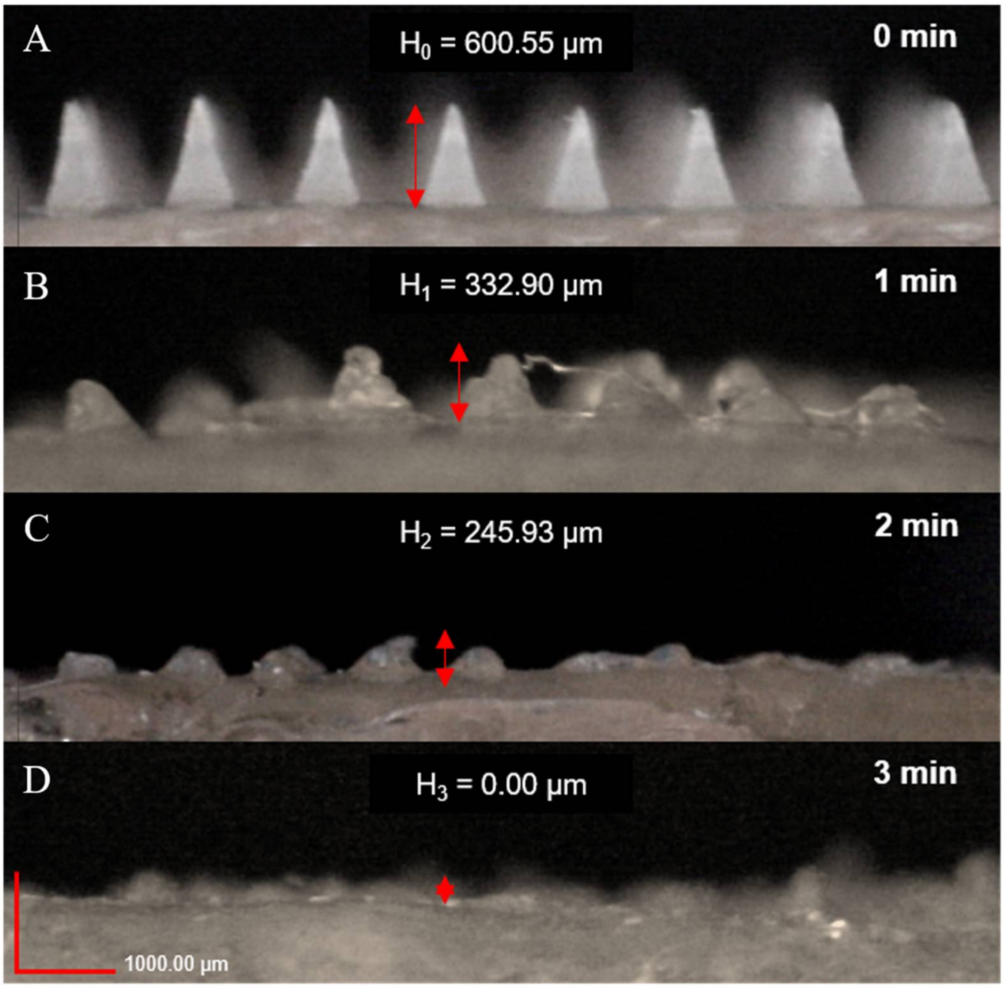

Figure S1. The dissolution times of MEs-FLUZ loaded two-layered dissolving MNs in corneal tissues at)A(0 min,)B(1 min,)C(2 min and (D) 3 min
